# MiR-21-5p modulates LPS-induced acute injury in alveolar epithelial cells by targeting SLC16A10

**DOI:** 10.1038/s41598-024-61777-x

**Published:** 2024-05-15

**Authors:** Huanan Zeng, Yuqing Zhou, Zhi Liu, Wei Liu

**Affiliations:** Emergency Department, The First Hospital of China Medical University, No.155 of North Street Nanjing, Heping District, Shenyang, 110001 Liaoning China

**Keywords:** ALI, LPS, Cell model, Inflammation, MiR-21-5p, SLC16A10, Cell biology, Drug discovery, Molecular biology, Molecular medicine

## Abstract

Sepsis is a systemic inflammatory response syndrome resulting from the invasion of the human body by bacteria and other pathogenic microorganisms. One of its most prevalent complications is acute lung injury, which places a significant medical burden on numerous countries and regions due to its high morbidity and mortality rates. MicroRNA (miRNA) plays a critical role in the body's inflammatory response and immune regulation. Recent studies have focused on miR-21-5p in the context of acute lung injury, but its role appears to vary in different models of this condition. In the LPS-induced acute injury model of A549 cells, there is differential expression, but the specific mechanism remains unclear. Therefore, our aim is to investigate the changes in the expression of miR-21-5p and SLC16A10 in a type II alveolar epithelial cell injury model induced by LPS and explore the therapeutic effects of their targeted regulation. A549 cells were directly stimulated with 10 µg/ml of LPS to construct a model of LPS-induced cell injury. Cells were collected at different time points and the expression of interleukin 1 beta (IL-1β), tumor necrosis factor-α (TNF-α) and miR-21-5p were measured by RT-qPCR and western blot. Then miR-21-5p mimic transfection was used to up-regulate the expression of miR-21-5p in A549 cells and the expression of IL-1β and TNF-α in each group of cells was measured by RT-qPCR and western blot. The miRDB, TargetScan, miRWalk, Starbase, Tarbase and miR Tarbase databases were used to predict the miR-21-5p target genes and simultaneously, the DisGeNet database was used to search the sepsis-related gene groups. The intersection of the two groups was taken as the core gene. Luciferase reporter assay further verified SLC16A10 as the core gene with miR-21-5p. The expression of miR-21-5p and SLC16A10 were regulated by transfection or inhibitors in A549 cells with or without LPS stimulation. And then the expression of IL-1β and TNF-α in A549 cells was tested by RT-qPCR and western blot in different groups, clarifying the role of miR-21-5p-SLC16A10 axis in LPS-induced inflammatory injury in A549 cells. (1) IL-1β and TNF-α mRNA and protein expression significantly increased at 6, 12, and 24 h after LPS stimulation as well as the miR-21-5p expression compared with the control group (P < 0.05). (2) After overexpression of miR-21-5p in A549 cells, the expression of IL-1β and TNF-α was significantly reduced after LPS stimulation, suggesting that miR-21-5p has a protection against LPS-induced injury. (3) The core gene set, comprising 51 target genes of miR-21-5p intersecting with the 1448 sepsis-related genes, was identified. This set includes SLC16A10, TNPO1, STAT3, PIK3R1, and FASLG. Following a literature review, SLC16A10 was selected as the ultimate target gene. Dual luciferase assay results confirmed that SLC16A10 is indeed a target gene of miR-21-5p. (4) Knocking down SLC16A10 expression by siRNA significantly reduced the expression of IL-1β and TNF-α in A549 cells after LPS treatment (P < 0.05). (5) miR-21-5p inhibitor increased the expression levels of IL-1β and TNF-α in A549 cells after LPS stimulation (P < 0.05). In comparison to cells solely transfected with miR-21-5p inhibitor, co-transfection of miR-21-5p inhibitor and si-SLC6A10 significantly reduced the expression of IL-1β and TNF-α (P < 0.05). MiR-21-5p plays a protective role in LPS-induced acute inflammatory injury of A549 cells. By targeting SLC16A10, it effectively mitigates the inflammatory response in A549 cells induced by LPS. Furthermore, SLC16A10 holds promise as a potential target for the treatment of acute lung injury.

## Introduction

After centuries of evolution, sepsis remains a substantial global public health concern^1^. Acute lung injury (ALI) is a commonly occurring complication of sepsis, leading to elevated rates of morbidity and mortality^2,3^. Notably, infection with LPS, Gram-negative bacterial endotoxin, is acknowledged as a significant factor contributing to the onset of acute lung injury in sepsis^4,5^.

LPS infiltrates the lungs and adheres to the lung surface, triggering lung inflammation^5^. Alveolar epithelial cells, particularly type II alveolar epithelial cells, act as the initial line of defense in this process. They engage in synthesizing and secreting essential substances^6^, contribute to the inflammatory response in ALI^7–9^, regulate coagulation and fibrinolysis within the alveoli^10–12^, assist in expelling fluid from the alveoli^13^, modulate the immune system^14^, and support the proliferation and repair of Type I alveolar epithelial cells^15^. Consequently, further research into the role and mechanisms of type II alveolar epithelial cells in ALI/ARDS is of paramount importance.

MicroRNA (miRNA), is a type of single-stranded non-coding RNA that is approximately 22 nucleotides in length^16^. It functions in post-transcriptional gene regulation. MiRNAs possess the ability to regulate gene expression at the post-transcriptional level by binding to specific messenger ribonucleic acids (mRNAs). Over the years, extensive research has been conducted on miRNAs in the field of tumors, revealing their involvement in the regulation of various biological functions such as aging, autophagy, apoptosis, and proliferation^17^. Additionally, miRNAs play a crucial role in inflammation and immune regulation.

MiR-21-5p, also referred to as miR-21, is a highly conserved species of miRNA. Several studies have been conducted on miR-21-5p in ALI^17^. The imbalance of the inflammatory response is considered a central theory in ALI, and miR-21-5p has been found to play a dual role in regulating this inflammatory response. In terms of pro-inflammatory effects, in LPS-induced ALI mouse models, miR-21-5p knockout has been shown to suppress the inflammatory response and promote mouse survival^18,19^. Conversely, in LPS-induced lung fibroblasts (WI-38)^20^, mouse lung microvascular endothelial cells (MPVECs)^21^, and human alveolar epithelial cells (HPAEpiC)^19^ models, overexpression of miR-21-5p leads to an increase in the release of IL-6 and IL-1β. On the anti-inflammatory front, in a rat sepsis model prepared using cecal ligation and puncture (CLP) method, miR-21-5p inhibitor significantly increases the concentrations of IL-6, IL-8, and TNF-α^20^. In LPS-stimulated mouse macrophage cell lines (RAW264.7)^22^ and lung macrophages (NR8383)^23^ models, upregulation of miR-21-5p can inhibit the expression of inflammatory factors.

MiR-21-5p exhibits a dual role, and its effects are cell-specific, exerting different functions in various cell models. To date, the role of miR-21-5p in LPS-induced alveolar epithelial cells (A549) has not been reported. In this study, our primary goal was to establish an LPS-induced acute injury model using A549 cells and investigate the mechanisms and changes in the expression of miR-21-5p together with the inflammatory factors. Simultaneously we used target gene databases and disease-related gene prediction websites to screen for the target genes of miR-21-5p. Ultimately, our findings will provide compelling evidence regarding the involvement and regulation of miR-21-5p in the inflammatory processes during ALI.

## Materials and methods

### Cell lines and cultures

The cell lines employed in this study included the human pulmonary epithelial cell line A549 (obtained from the Cell Bank of Shanghai Institute of Biochemistry and Cell Biology, Shanghai, China) and HEK 293T (acquired from the Cell Bank of the Chinese Academy of Sciences, Shanghai, China). All cell lines were confirmed to be free of mycoplasma infection.

A549 cells were cultured in DMEM/F12 medium (MA0214-2, Meilun, China), while HEK 293T cells were cultured in DMEM medium (SC102-02, Seven, China). Both media were supplemented with 10% fetal bovine serum (FBS, AusGeneX, Australia) and 1% penicillin–streptomycin (PWL062, Meilun, China). The cells were maintained at 37 °C in a humidified atmosphere with 5% CO_2_.

To investigate the functional role of miR-21-5p in acute lung injury, A549 cells were exposed to 10 μg/mL LPS (ST1470, Biyuntian, China) or subjected to other relevant treatments. Subsequent testing of these cells was conducted after the specified treatments.

### Cell transfection

Cell transfection was carried out using synthesized miR-21-5p mimic or inhibitor, SLC16A10 siRNA (si-SLC16A10), as well as their respective negative controls (NC) provided by GenePharma (Shanghai, China). Lipofectamine 3000 (Invitrogen, USA) was used for transfecting the cells with a final concentration of 100 nM, following the manufacturer's instructions.

A549 cells were cultured in 6-well plates in antibiotic-free medium at a concentration of 5 × 10^4^ cells/mL one day before transfection. Subsequently, the cells were randomly divided into 14 groups, as follows: SLC16A10 siRNA, NC siRNA, miR-21-5p mimic, NC mimic, miR-21-5p inhibitor, NC inhibitor, SLC16A10 siRNA + LPS, NC siRNA + LPS, miR-21-5p mimic + LPS, NC mimic + LPS, miR-21-5p inhibitor + LPS, NC inhibitor + LPS, miR-21-5p inhibitor + NC siRNA + LPS, and miR-21-5p inhibitor + SLC16A10 siRNA + LPS.

When A549 cells reached 50–70% confluence, Lipofectamine 3000 reagent (Invitrogen, USA) and serum-free medium were utilized to perform the transfection, following the manufacturer's instructions. After 6 h, the transfection medium was replaced with fresh growth medium, and the cells were further cultured for an additional 48 h. The transfection efficiency was then assessed using quantitative real-time RT-qPCR. The cells were either collected for analysis or further cultured in the presence of 10 μg/mL lipopolysaccharide for 6 or 24 h, following which the A549 cells were collected for further analysis. The sequences of siRNA, microRNA mimic, and microRNA inhibitor used in this study are listed in Table 1.

**Table 1:** RNA sequence.

Name		Sequence
NC mimic	Sense	5′-UUCUCCGAACGUGUCACGUTT-3′
	Antisense	5′-ACGUGACACGUUCGGAGAATT-3
Hsa-miR-21-5p mimic	Sense	5′-UAGCUUAUCAGACUGAUGUUGA-3′
	Antisense	5′-AACAUCAGUCUGAUAAGCUAUU-3′
NC inhibitor	Sense	5′-CAGUACUUUUGUGUAGUACAA-3′
Hsa-miR-21-5p inhibitor	Sense	5′-UCAACAUCAGUCUGAUAAGCUA-3′
NC siRNA	Sense	5′-UUCUCCGAACGUGUCACGUTT-3
	Antisense	5′-ACGUGACACGUUCGGAGAATT-3′
SLC16A10 siRNA	Sense	5′-GCGUCUUCACAGACCUAUUTT-3′
	Antisense	5′-AAUAGGUCUGUGAAGACGCTT-3′

### Bioinformatics

The potential target genes of miR-21-5p were predicted using various online tools, including TargetScan Human 8.0 (http://www.targetscan.org/), miRDB (2010.8) (http://mirdb.org/miRDB/), miRWalk3.0 (http://mirwalk.umm.uni-heidelberg.de/), starBase v2.0 (http://starbase.sysu.edu.cn/), and TarBase v.8 (http://microrna.gr/tarbase/). Additionally, we utilized the DisGeNet V7.0 database (https://www.disgenet.org/) to explore genes associated with sepsis.

### Plasmids construction and luciferase reporter assay

Wild-type (wt) and mutant-type (mut) luciferase reporter plasmids, psiCHECK2-SLC16A10-3′-UTR, were generated by inserting the SLC16A10 mRNA 3′‐UTR sequences containing the binding site into the psiCHECK2 plasmids. HEK293T cells were co-transfected with luciferase reporter plasmids and either miR-21-5p mimic or NC mimic using Lipofectamine 3000 (Invitrogen, USA), following the manufacturer's instructions. For the luciferase reporter assay, the luciferase activities were measured 48 h after transfection using the Dual‐Luciferase Reporter Assay System (E1910, Promega, USA), following the manufacturer's protocol.

### Quantitative real-time PCR (qPCR)

RNA samples were extracted from A549 cells using the AG RNAex Pro kit (AG21102, Accurate, China) in accordance with the manufacturer's protocol. The concentration and purity of the RNA were determined using the NanoDrop™ 2000 spectrophotometer (Thermo Scientific™, USA). The OD260/280 ratios for all samples fell within the range of 1.9–2.1. One microgram of total RNA was reverse transcribed into cDNA using the Evo-M-MLV RT Kit with gDNA Clean for qPCR (AG11705, Accurate, China) following the manufacturer's protocol. Subsequently, qPCR was conducted using the SYBR GREEN Premix Pro Taq HS qPCR Kit (AG11701, Accurate, China) as per the manufacturer's instructions and the Light Cycle 96 Instrument (Roche Life Science, Canada). The following thermocycling conditions were applied for qPCR: initial denaturation at 95 °C for 30 s, followed by 40 cycles of 15 s at 95 °C and 30 s at 60 °C. The forward and reverse primer sequences for RT-qPCR of each gene were as listed in Table 2.

**Table 2:** Primer sequence for qPCR.

Gene		Primer sequence
Hsa-miR-21-5p	Forward	5′-GCGCGTAGCTTATCAGACTGA-3′
	Reverse	5′-AGTGCAGGGTCCGAGGTATT-3′
	RT-primer	5′-GTCGTATCCAGTGCAGGGTCCGAGGTATTCGCACTGGATACGACTCAACA-3′
Hsa-IL-1β	Forward	5′-GATCACTGAACTGCACGCTCC-3′
	Reverse	5′-ACTTGTTGCTCCATATCCTGT-3′
Hsa-TNF-α	Forward	5′-TTCTCCTTCCTGATCGTGG-3′
	Reverse	5′-AAGATGATCTGACTGCC-3′
Hsa-SLC16A10	Forward	5′-TTTACTTACCGACCTCTTGCT-3′
	Reverse	5′-TCACCTTGAAGATGGCAAA-3′
GAPDH	Forward	5′-ACGGATTTGGTCGTATTGG-3′
	Reverse	5′-TCCCGTTCTCAGCCTTG-3′
U6	Forward	5′-GGAACGATACAGAGAAGATTAGC-3′
	Reverse	5′-TGGAACGCTTCACGAATTTGCG-3′

The expression of SLC16A10/IL-1β/TNF-α was normalized to GAPDH, and the expression of miR-21-5p was normalized to U6. Gene expression was calculated using the 2^−ΔΔCt^ method. Each experiment was independently repeated three times.

### Western blotting

Total protein was extracted from A549 cells using RIPA lysis buffer (P0013B, Beyotime, China) containing PMSF (ST506, Beyotime, China). The total protein concentration was quantified using a BCA assay kit (P0010S, Beyotime, China). A total of 20 µg of protein per lane was separated by 15% SDS-PAGE (KGP113, Kegen, China) and transferred onto 0.22 PVDF membranes (No162-0.177, Bio-Rad, America). After identifying the location of the target band based on the protein marker, excise the gel, and cut a suitably sized piece of PVDF membrane. Transfer for 30 min if the molecular weight is below 20 kDa (IL-1β and TNF-α), and for 1 h if it is above 20 kDa (SLC16A10 and GAPDH).

The PVDF membranes were blocked with 5% non-fat milk (P0216, Beyotime China) in TBS-Tween20 (T1081, Slarbio China) for 2 h at room temperature. Subsequently, they were incubated overnight at 4 °C with primary antibodies diluted in TBS-Tween20: Anti-SLC16A10 antibody (YT7177, Immunoway, America) diluted 1:1000, Anti-IL-1β antibody (YT5201, Immunoway, America) diluted 1:1000, and Anti-TNF-α antibody (YT4689, Immunoway, America) diluted 1:1000. A 1:10,000 dilution of monoclonal anti-GAPDH antibody produced in mouse (60004-1-Ig, proteintech, China) was used as the loading control.

The following day, the membranes were washed and incubated for 1 h with secondary antibodies: goat-anti-rabbit IgG HRP (SA00001-2, proteintech, China) or goat-anti-mouse IgG HRP (SA00001-1, proteintech, China), both diluted 1:10,000. Protein signals were detected using Clarity western ECL substrate (No170506, Bio-Rad, America) and quantified with ImageJ v.2.0 software (National Institutes of Health). The relative protein expression was determined by calculating the ratio of the signals from the protein of interest to GAPDH. Each experiment was independently repeated at least three times under the same experimental conditions.

### Statistical analysis

All data in this study underwent statistical analysis using SPSS 26.0 (SPSS, Chicago), and the results were visually presented using GraphPad Prism v8.0 (GraphPad Software, Inc.). Values were expressed as the mean ± standard deviation (SD). For group sizes of 4 or less, one-way analysis of variance (ANOVA) was employed to compare qPCR and western blot results between groups, followed by post-hoc tests to determine significance. If variances were equal, the least significant difference (LSD) method was used, and if variances were unequal, Tamhane’s T2 method was applied. A p-value of less than 0.05 was considered statistically significant. For group sizes greater than 4, t-tests were used to compare qPCR and western blot results between groups, and a p-value of less than 0.025 was considered statistically significant.

## Results

### Establishment of an LPS-induced A549 cell injury model and evaluation of changes in inflammatory factor expression

**Figure 1:**
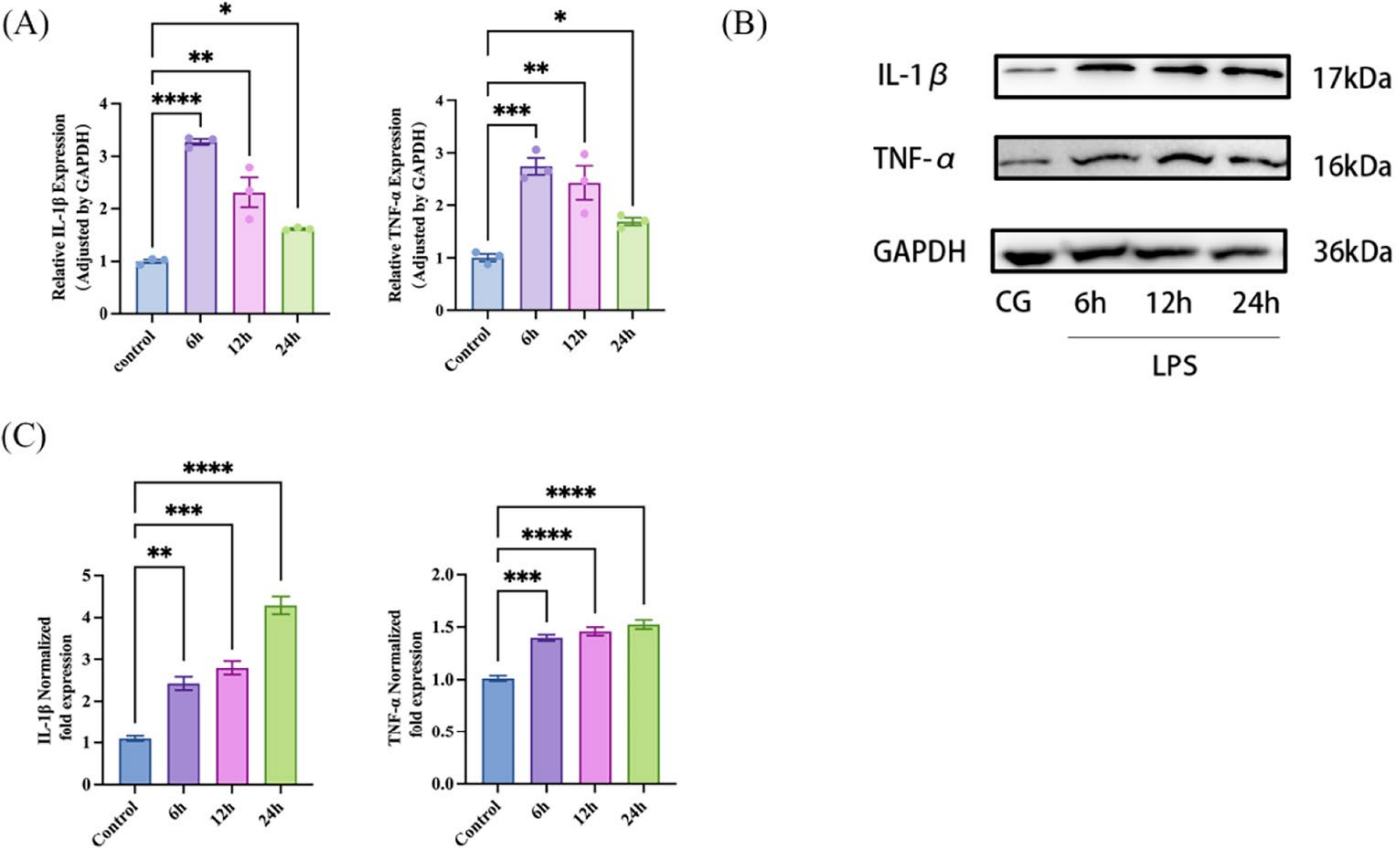
IL-1β and TNF-α expression in A549 cells are upregulated along with LPS-treated. The mRNA levels of IL-1β and TNF-α were detected by RT-qPCR in four groups in A549 cells including the control group and LPS treatment group for 6 h,12 h and 24 h (n = 3). Western blot further verified the expression levels of IL-1β and TNF-α in four groups. Quantification analysis of protein expression levels of IL-1β and TNF-α (n = 3). All data are shown as the mean ± SEM. ****P ≤ 0.0001, ***P ≤ 0.001, **P < 0.01, *P < 0.05; NS, not significant (P > 0.05).

To establish an acute injury model of alveolar epithelial cells, we exposed the cells to 10 µg/mL of LPS and assessed the expression of pro-inflammatory factors, IL-1β and TNF-α, at 6, 12, and 24-h time points. As illustrated in (Fig. 1A–C), the mRNA and protein expression levels of pro-inflammatory factors (IL-1β and TNF-α) were significantly elevated in the LPS-treated cells when compared to the control group (P < 0.05), confirming the success of the model establishment. It's noteworthy that in A549 cells subjected to LPS treatment at each time point, the mRNA expression of pro-inflammatory factors IL-1β and TNF-α reached their peaks at 6 h (Fig. 1A), while protein expression peaked at 24 h (Fig. 1B,C).

In order to ensure consistent and stable expression of inflammatory factors and facilitate subsequent experiments to observe changes in their expression, we chose to assess mRNA levels 6 h after LPS treatment and protein levels 24 h later.

### Characteristics of miR-21-5p expression alterations following LPS stimulation in A549 cells

**Figure 2:**
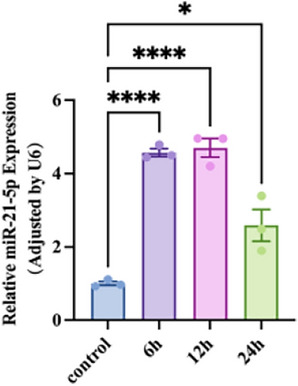
miR-21-5p expression in A549 cells are upregulated along with LPS-treated. The mRNA levels of IL-1β and TNF-α were detected by RT-qPCR in four groups in A549 cells including the control group and LPS treatment group for 6 h,12 h and 24 h (n = 3). All data are shown as the mean ± SEM. ****P ≤ 0.0001, ***P ≤ 0.001, **P < 0.01, *P < 0.05; NS, not significant (P > 0.05).

To investigate the expression of miR-21-5p in an LPS-induced acute lung injury model, we assessed the mRNA expression of miR-21-5p in A549 cells after 6, 12, and 24 h of LPS treatment. As illustrated in (Fig. 2), in comparison to the control group, the mRNA expression of miR-21-5p in LPS-treated cells exhibited a gradual increase at 6 and 12 h, followed by a decrease at 24 h. Nevertheless, even at the 24-h time point, the expression level remained higher than that of the control group.

### MiR-21-5p overexpression decreased the inflammatory factors levels in LPS induced A549 cells

**Figure 3:**
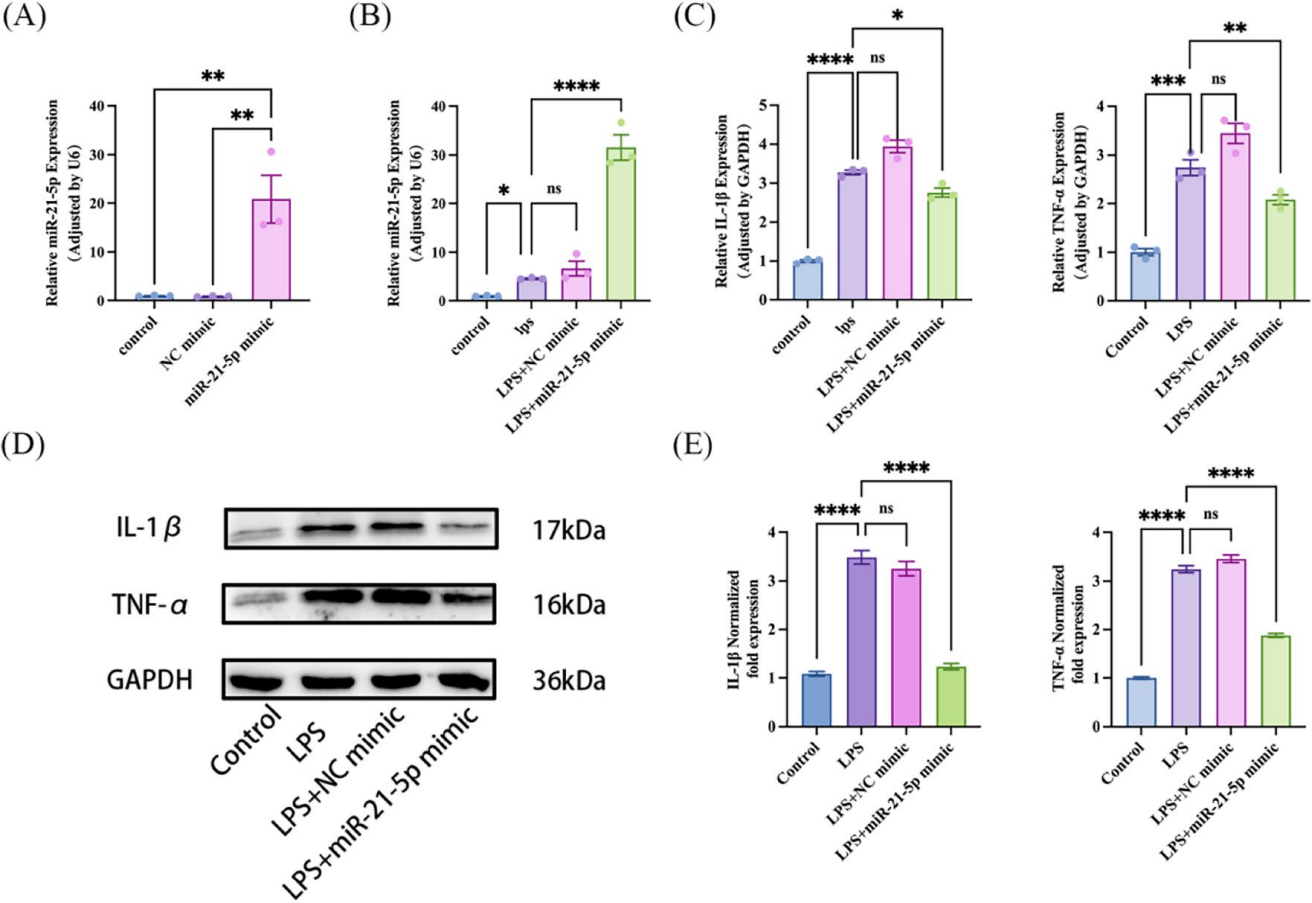
Overexpression of miR-21-5p can improve the inflammatory response induced by LPS-induced A549 cells. (**A**) A549 cells were transfected with miR-21-5p mimic, the mRNA levels of miR-21-5p was detected by RT-qPCR in three groups including the control group, NC mimic and miR-21-5p mimic (n = 3). (**B**) A549 cells were transfected with miR-21-5p mimic, followed by exposure to LPS, the mRNA levels of miR-21-5p was detected by RT-qPCR in four groups including the control group, LPS, LPS + NC mimic and LPS + miR-21-5p mimic (n = 3). (**C**) The mRNA levels of IL-1β and TNF-α were further confirmed by RT-qPCR in four groups. (n = 3). (**D**) Western blot further verified the expression levels of IL-1β and TNF-α in four groups. (**E**) Quantification analysis of protein expression levels of IL-1β and TNF-α (n = 3). All data are shown as the mean ± SEM. ****P ≤ 0.0001, ***P ≤ 0.001, **P < 0.01, *P < 0.05; NS, not significant (P > 0.05).

To assess the impact of miR-21-5p on the inflammatory response in acute lung injury, we investigated the levels of inflammatory factor mRNA and protein in cells treated with a miR-21-5p mimic. A549 cells were transfected with miR-21-5p mimics for 48 h. The qPCR results clearly indicated a significant increase in miR-21-5p mRNA expression when compared to the control group or the NC mimic group, affirming the successful transfection (Fig. 3A). Furthermore, upon exposure to LPS for 6 h, qPCR results demonstrated a further elevation in miR-21-5p mRNA expression, indicating the stability and reliability of the transfection model (Fig. 3B). This model can be effectively employed in subsequent experimental investigations.

A549 cells were transfected with the miR-21-5p mimic for 48 h, followed by a 6-h exposure to LPS, and the expression of IL-1β and TNF-α was analyzed using qPCR. As presented in (Fig. 3C), the overexpression of miR-21-5p through the miR-21-5p mimic significantly reduced the production of IL-1β and TNF-α induced by LPS. Moreover, after exposure to LPS for 24 h following transfection, western blot results demonstrated that the protein levels of IL-1β and TNF-α, which were up-regulated by LPS, were markedly suppressed by the overexpression of miR-21-5p (Fig. 3D,E). The findings indicate that miR-21-5p plays a negative regulatory role in the inflammatory response during LPS-induced acute lung injury.

### Predicted target genes of miR-21-5p

**Figure 4:**
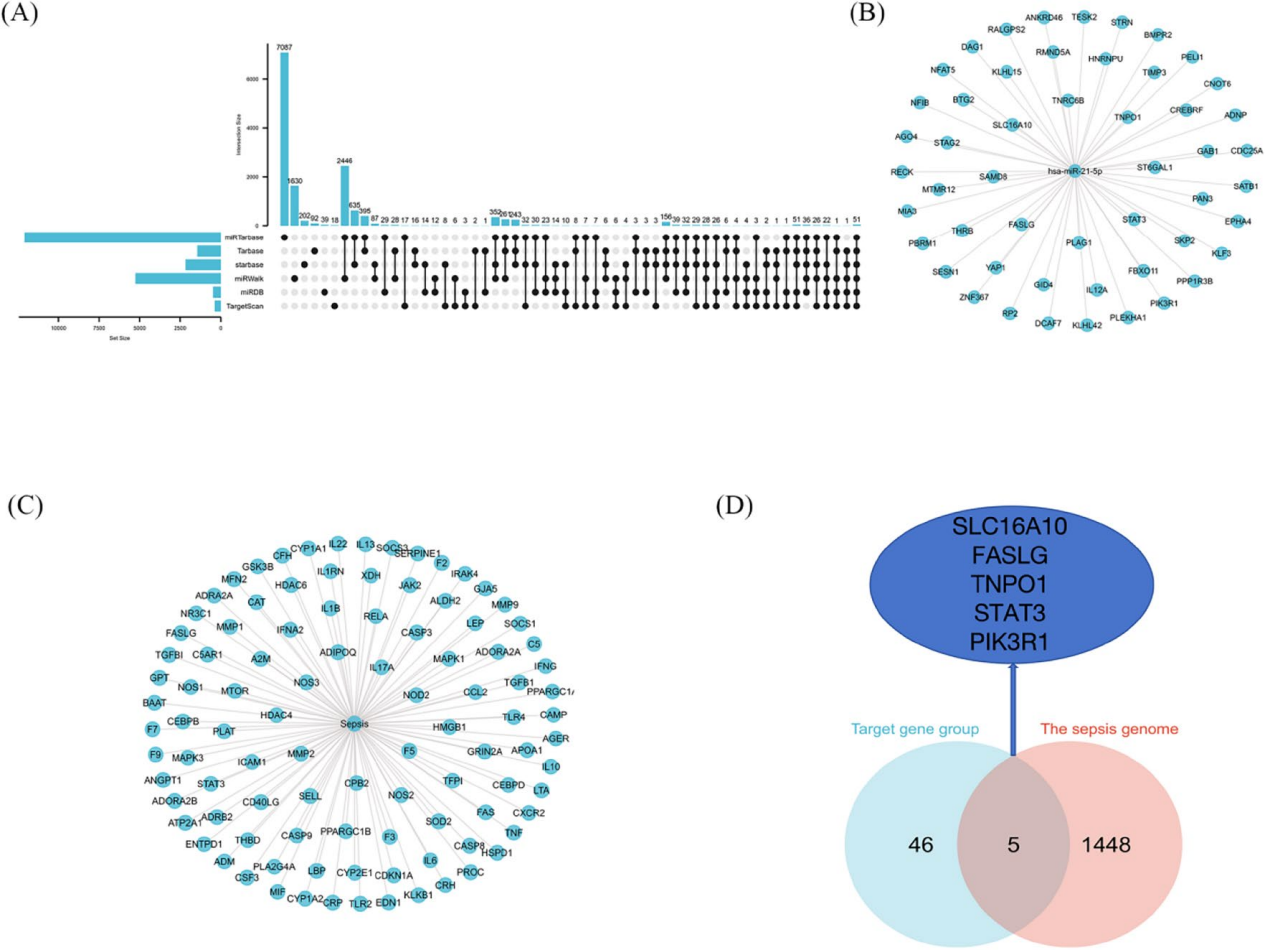
Prediction of miR-21-5p target gene. (**A**) The distribution of miR-21-5p target genes in the prediction software was shown by upset diagram, including miRDB, TargetScan, miRWalk, Starbase, Tarbase, Mir-tarbase, and the number of intersecting target genes. (**B**) The above six databases are interlinked, specifically containing target genes. (**C**) Some of the genes associated with sepsis predicted in DisGeNet database. (**D**) The Venn diagram shows the intersection of genes associated with sepsis and target genes.

To investigate the regulatory role of miR-21-5p in the inflammatory response and identify potential target genes, this study employed six widely recognized miRNA target gene prediction databases (miRDB, TargetScan, miRWalk, Starbase, Tarbase, miRTarbase). A total of 51 target genes were consistently identified across all databases, as illustrated in (Fig. 4A). The specific genes are listed in (Fig. 4B). Additionally, the DisGeNet database was utilized to search for sepsis-related genes, resulting in a total of 1453 genes. The top 100 genes, ranked by Score in descending order, are presented in (Fig. 4C). Upon data analysis, it was determined that there is a total of 5 genes that intersect between these datasets. These genes include SLC16A10, TNPO1, FASLG, STAT3, and PIK3R1 (Fig. 4D). Detailed bioinformatics data can be seen in Supplementary 1.

After conducting a comprehensive review of the relevant literature, it has been established that the targeting relationship between TNPO1, FASLG, STAT3, PIK3R1, and miR-21 has been confirmed in several disease models. For more detailed information, please refer to Table 3. Previous studies have indicated that miRNA evolution is relatively conservative, and the relationship between target genes and miRNA targets is not species-specific^24^.

**Table 3:** Literature review of relevant target genes.

Author and reference	MiRNA	Target genes	Year of publication	Disease
Zhai et al.^41^	miR-21	TNPO1	2019	Hair follicle development
Du et al.^42^	miR-21	FASLG	2019	Deep venous thrombosis
Yang et al.^43^	miR-21	FASLG	2014	Stomach neoplasm
Shang et al.^44^	miR-21	FASLG	2015	Spongioblastoma glioblastoma
Chen et al.^45^	miR-21	FASLG	2019	Hepatocellular carcinoma
Yan et al.^46^	miR-21	PIK3R1	2016	Breast cancer
Tang et al.^47^	miR-21	FASLG	2019	Myocardial ischemia–reperfusion injury
Du et al.^22^	miR-21	STAT3	2019	Acute lung injury
Fang et al.^48^	miR-21	STAT3	2020	Cervical cancer
Li et al.^49^	miR-21	STAT3	2021	Osteosarcoma
Li et al.^50^	miR-21	STAT3	2020	Idiopathic arthritis
Francis et al.^51^	miR-21	STAT3	2014	Alcoholic liver injury
Lu et al.^52^	miR-21	STAT3	2020	Ulcerative colitis

SLC16A10, a member of the SLC16 gene family, functions as an aromatic amino acid transporter^25^. Its primary role is to transport aromatic amino acids independently of sodium and protons. In recent years, it has been discovered that SLC16A10 plays a role in immune regulation and influences tumor development. Research has shown a significant increase in the expression of SLC16A10 in patients with esophageal cancer, which has a negative impact^26,27^. Furthermore, recent studies have demonstrated the involvement of SLC16A10 in the body's inflammatory response process^28^.

Considering the role of SLC16A10 in immune regulation and the inflammatory response, it is important to note that there is currently no research available on its involvement in the ALI model. Furthermore, the potential targeting relationship between miR-21 and SLC16A10 has not been verified. Therefore, this study focuses on investigating SLC16A10 as the research target.

### Validation of miR-21-5p target genes

**Figure 5:**
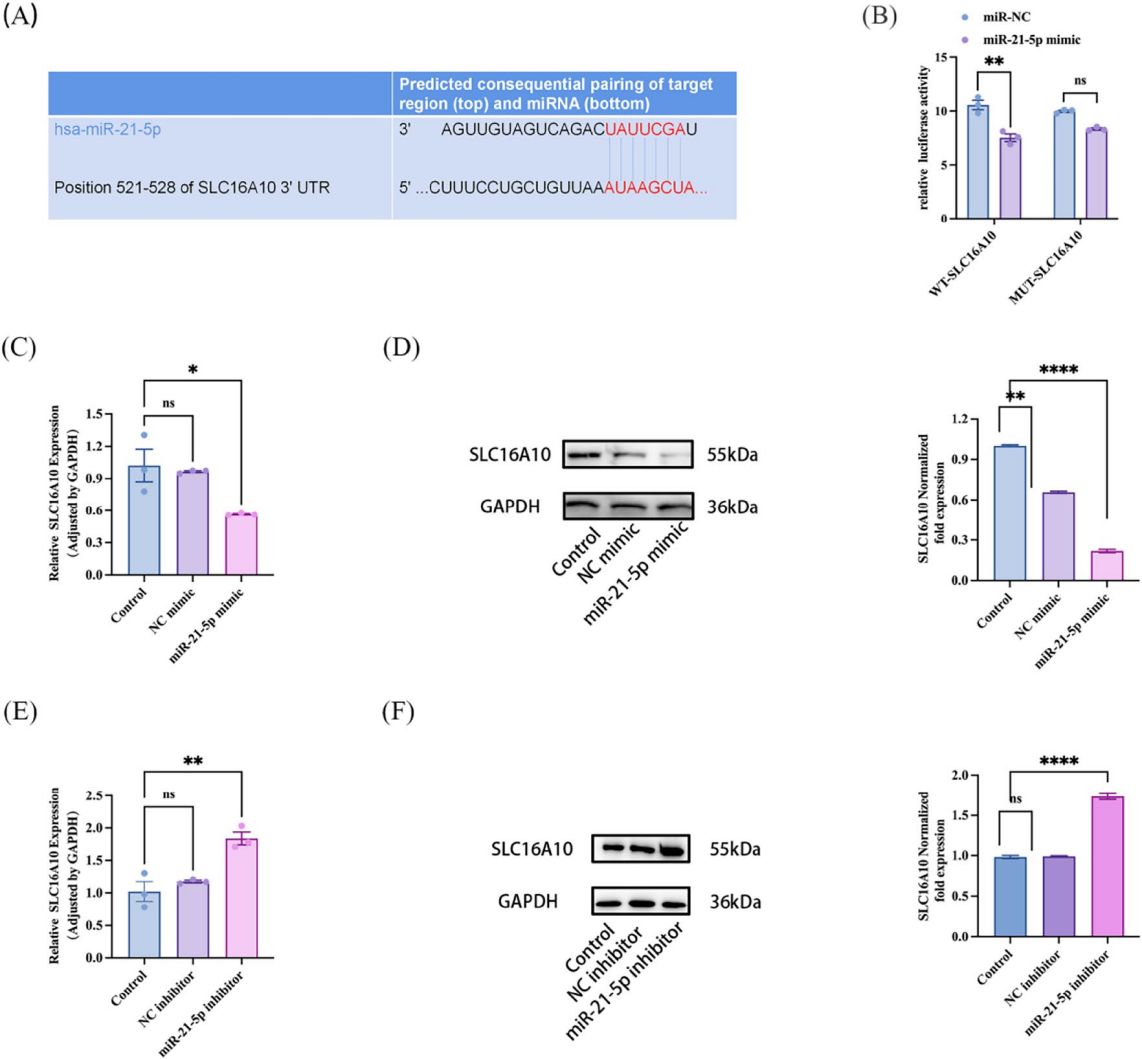
SLC16A10 is a target gene of miR-21-5p. (**A**) TargetScan predict the binding site of miR-21-5p to SLC16A10-3′UTR region. (**B**) Double luciferase report Method to detect the binding effect of miR-21-5p with SLC16A10-3′UTR and mutant region 3′ UTR. (**C**) A549 cells were transfected with miR-21-5p mimic, The SLC16A10 expression was detected by RT-qPCR. (n = 3). (**D**) Western blot detection and quantification of SLC16A10 protein expression level (n = 3). A549 cells were transfected with miR-21-5p mimic. GAPDH was defined as internal control. (**E**) A549 cells were transfected with miR-21-5p inhibitor, The SLC16A10 expression was detected by RT-qPCR. (n = 3). (**F**) Western blot detection and quantification of SLC16A10 protein expression level (n = 3). A549 cells were transfected with miR-21-5p inhibitor. GAPDH was defined as internal control. All data are shown as the mean ± SEM. ****P ≤ 0.0001, ***P ≤ 0.001, **P < 0.01, *P < 0.05; NS, not significant (P > 0.05).

To investigate whether SLC16A10 is the target gene of miR-21-5p, we utilized the TargetScan website to predict the binding site between miR-21-5p and SLC16A10 (Fig. 5A). Additionally, we generated the SLC16A10 transfection plasmid. Subsequently, we co-transfected SLC16A10-WT or SLC16A10-MUT along with miR-21-5p mimic or NC mimic into 293 T cells for 48 h. Finally, we assessed the luciferase activity using the dual luciferase reporter assay. The detection report revealed that the luciferase activity in cells co-transfected with SLC16A10-WT and miR-21-5p mimic was significantly inhibited compared to the co-transfection of SLC16A10-WT and NC mimic. In comparison, luciferase activity remained largely unchanged in cells co-transfected with SLC16A10-MUT and miR-21-5p mimic, as opposed to those co-transfected with SLC16A10-MUT and NC mimic (Fig. 5B).

To further verify the direct targeting relationship between the two, this experiment examined the expression of SLC16A10 in A549 cells after transfecting them with NC mimic or miR-21-5p mimic for 48 h. The results showed that compared to the NC mimic group, overexpression of miR-21-5p significantly reduced the mRNA and protein expression of SLC16A10 (Fig. 5C,D). 48 h after transferring NC inhibitor or miR-21-5p inhibitor into A549 cells, we detected the expression of SLC16A10. We observed a significant increase in the expression of SLC16A10 at the mRNA and protein levels when miR-21-5p was knocked down, compared to the empty inhibitor control group (Fig. 5E,F). The above results fully confirm that miR-21-5p directly targets SLC16A10.

### Knocking-down SLC16A10 suppressed the production of inflammatory factors in A549 cells

**Figure 6:**
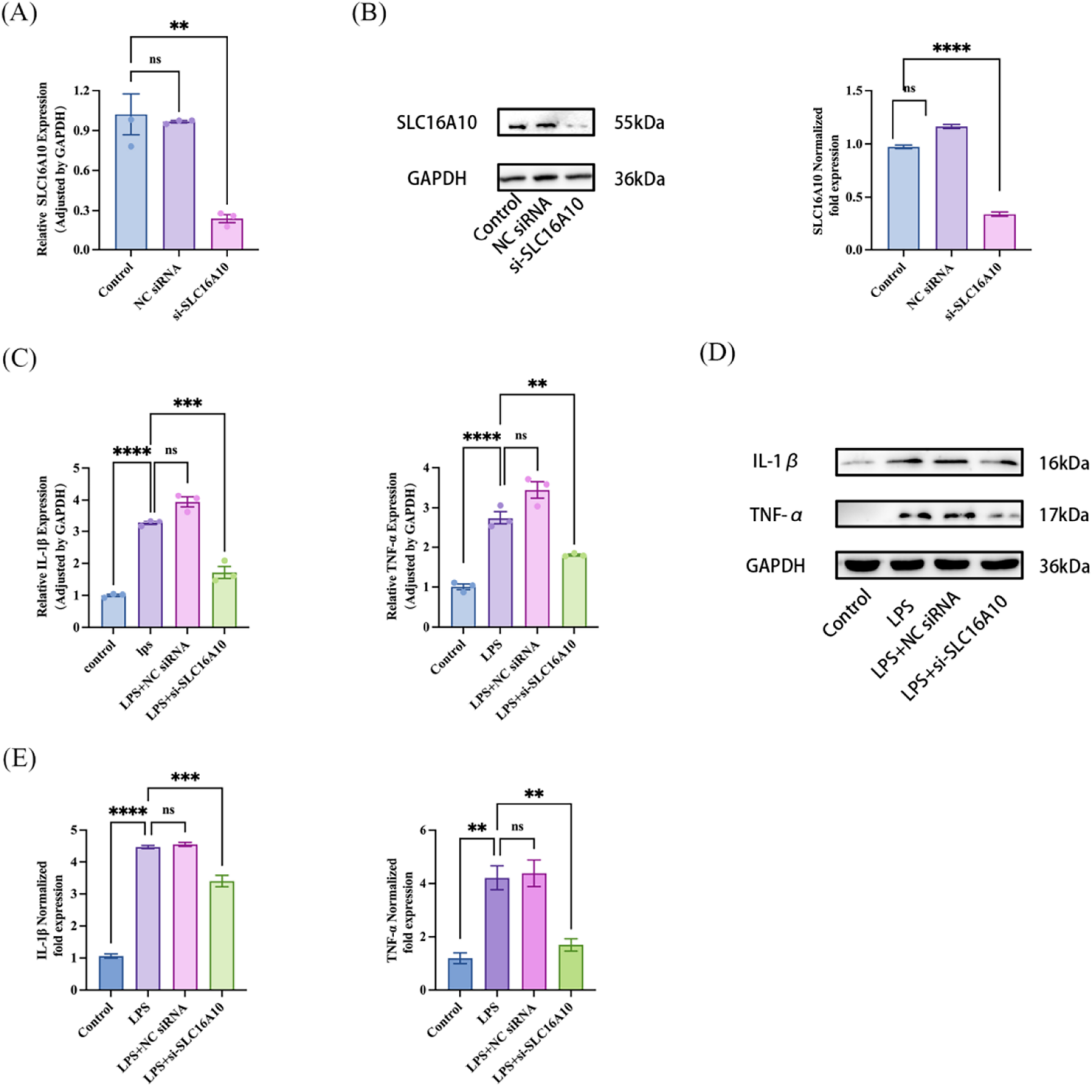
Knockdown of SLC16A10 can improve the LPS-induced inflammatory response of A549 cells. (**A**) A549 cells were transfected with SLC16A10 siRNA, the mRNA levels of SLC16A10 was detected by RT-qPCR in three groups including the control group, NC siRNA and si-SLC16A10 (n = 3). (**B**) Western blot detection and quantification of SLC16A10 protein expression level (n = 3). A549 cells were transfected with SLC16A10 siRNA. GAPDH was defined as internal control. (**C**) A549 cells were transfected with SLC16A10 siRNA, followed by exposure to LPS, the mRNA levels of IL-1β and TNF-α was detected by RT-qPCR in four groups including the control group, LPS, LPS + NC siRNA and LPS + si-SLC16A10 (n = 3). (**D**) Western blot further verified the expression levels of IL-1β and TNF-α in four groups. (**E**) Quantification analysis of protein expression levels of IL-1β and TNF-α (n = 3). All data are shown as the mean ± SEM. ****P ≤ 0.0001, ***P ≤ 0.001, **P < 0.01, *P < 0.05; NS, not significant (P > 0.05).

To inhibit the expression of SLC16A10 in A549 cells, we examined the mRNA and protein levels of SLC16A10 in A549 cells treated with SLC16A10 siRNA. According to (Fig. 6A,B), the mRNA and protein expression of SLC16A10 were observed to be reduced in SLC16A10 siRNA-treated A549 cells, in comparison to both the control group and the A549 cells treated with non-specific siRNA. The results indicated that transient transfection of si-SLC16A10 successfully down-regulated the level of SLC16A10 in A549 cells.

To investigate the impact of SLC16A10 on the inflammatory response of A549 cells induced by LPS, we examined the mRNA and protein expression levels of inflammatory factors in A549 cells. These cells were treated with SLC16A10 siRNA and then induced by LPS. Following a 6-h exposure to LPS, the qPCR results indicated a significant increase in the mRNA expression levels of IL-1β and TNF-α in the LPS group compared to the control group. Furthermore, the SLC16A10 siRNA + LPS treatment group exhibited a significant decrease in the mRNA expression levels of IL-1β and TNF-α when compared to the NC siRNA + LPS treatment group (Fig. 6C).

Following a 24-h exposure to LPS, the WB results revealed a notable increase in the expression of IL-1β and TNF-α proteins in the LPS group cells compared to the control group. However, this effect was reversed when SLC16A10 knockdown was performed. In comparison to the NC siRNA + LPS group, the siRNA + LPS group exhibited significantly reduced protein expression levels of IL-1β and TNF-α due to SLC16A10 (Fig. 6D,E). The findings suggest that reducing the activity of SLC16A10 can potentially suppress the production of inflammatory factors in A549 cells when exposed to LPS.

### Validation of miR-21-5p regulation in LPS-induced inflammatory response in A549 cells by targeting SLC16A10

The above experimental results have confirmed that both overexpression of miR-21-5p and suppression of SLC16A10 expression can alleviate the inflammatory damage caused by LPS to A549 cells (Figs. 3D, 6D). MiR-21-5p has a targeted regulatory relationship with SLC16A10, meaning that overexpression of miR-21-5p inhibits SLC16A10 expression, while inhibiting miR-21-5p expression can elevate SLC16A10 expression (Fig. 5C–F).

**Figure 7:**
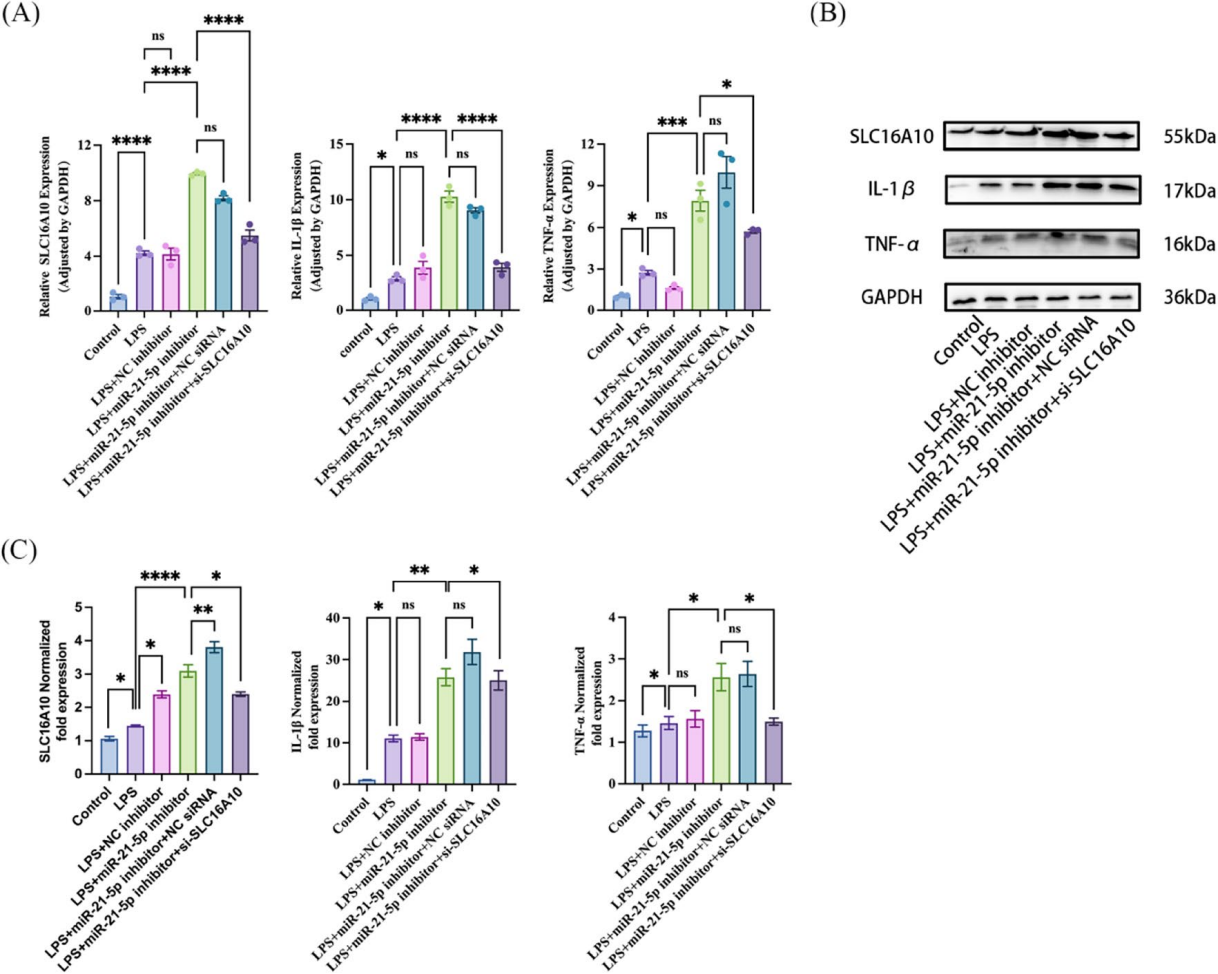
SLC16A10 mediates the anti-inflammatory effect of miR-21-5p. (**A**) A549 cells were co-transfected with miR-21-5p inhibitor and si-SLC16A10, and the mRNA levels of SLC16A10, IL-1β and TNF-α were measured by RT-qPCR in six groups. including the control group, LPS, LPS + NC inhibitor, LPS + miR-21-5p inhibitor, LPS + miR-21-5p inhibitor + NC siRNA, LPS + miR-21-5p inhibitor + si-SLC16A10 (n = 3). (**B**) Western blot further verified the expression levels of SLC16A10, IL-1β and TNF-α in six groups. (**C**) Quantification analysis of protein expression levels of SLC16A10, IL-1β and TNF-α (n = 3). All data are shown as the mean ± SEM. ****P ≤ 0.0001, ***P ≤ 0.001, **P < 0.01, *P < 0.05; NS, not significant (P > 0.05).

To further validate whether miR-21-5p plays a downstream regulatory role in the process of inflammatory damage in A549 cells induced by LPS by regulating SLC16A10, we separately and simultaneously transfected A549 cells with miR-21-5p inhibitor and SLC16A10 siRNA. After 48 h, the cells were stimulated with 10 µg/mL LPS, and the mRNA and protein expression levels of SLC16A10, IL-1β, and TNF-α were measured at 6 h and 24 h for each group. As shown in (Fig. 7), knocking down miR-21-5p to mitigate the inflammatory damage caused by LPS to A549 cells is reversed when SLC16A10 is concurrently knocked down. This suggests that in the LPS-induced A549 cell damage model, the low expression of miR-21-5p exerts a pro-inflammatory damage effect through the downstream regulation of SLC16A10. SLC16A10 holds promise as a novel target for mitigating inflammatory responses.

## Discussion

In this study, we present a novel discovery indicating that miR-21-5p might exert a protective effect against LPS-induced inflammatory injury by targeting SLC16A10, thereby efficiently reducing the expression of inflammatory cytokines.

LPS is a major component of the Gram-negative bacterial cell wall and is widely recognized as the most commonly used inducer in ALI cell models^5^. IL-1β and TNF-α are two key pro-inflammatory factors in the process of ALI and are also considered as one of the standards for the successful modeling of ALI/ARDS^29^. In this experiment, we first confirmed the LPS-induced acute lung injury model, which exhibited a significant increase in the expression of IL-1β and TNF-α when simulating ALI. This result provides a solid foundation for our subsequent experiments.

miR-21-5p is a mature miRNA located on human chromosome 17q23.2, with the sequence 5′-UAGCUUAUCAGACUGAU-3′. It plays a role in post-transcriptional gene regulation and is involved in the regulation of various phenotypes, including apoptosis, autophagy, proliferation, fibrosis, and aging^30^. While recent research has explored the role of miR-21-5p in ALI, its expression and effects vary across different models^16^. Furthermore, there is limited research on how miR-21-5p's targeted interference impacts inflammatory responses. In our study, we observed an upregulation of miR-21-5p in A549 cells following LPS treatment, particularly at 6 and 12 h, with sustained elevation at 24 h compared to the control group. Some studies suggest that the increased expression of miR-21-5p in response to LPS stimulation may be related to the presence of NF-κB and activator protein-1 (AP-1) binding sites within the miR-21-5p promoter region^31,32^. This implies that miR-21-5p may play a regulatory role in ALI, which has piqued our interest for further investigation.

Furthermore, through experimental validation, we found that the overexpression of miR-21-5p can inhibit the expression of inflammatory cytokines (IL-1β and TNF-α) induced by LPS, while the inhibition of miR-21-5p significantly increases the expression of these inflammatory factors. This suggests that miR-21-5p plays a negative regulatory role in the inflammatory response in our ALI model. There are reports that in an LPS-stimulated lung macrophage (NR8383) injury model, overexpression of miR-21-5p can reduce the expression of TNF-α and IL-1β in the experimental group, and it was found that miR-21-5p primarily targets TLR4 to inhibit NF-κB, resulting in reduced secretion of pro-inflammatory cytokines^23^. Another study reported that in an LPS-stimulated mouse macrophage (RAW264.7) model, miR-21-5p targets PDCD4 to inhibit NF-κB activation and reduce the production of inflammatory cytokines^33,34^. Similarly, in an infectious preterm labor model established in mouse placental trophoblast cells (MPCTs) stimulated by LPS, researchers found that miR-21-5p can directly target NF-κB to inhibit the production of inflammatory cytokines^35^. Taken together with our own research and that of others, these results suggest that miR-21-5p can regulate multiple target genes to inhibit the NF-κB pathway, thereby reducing the production of pro-inflammatory cytokines.

To further explore the target genes of miR-21-5p and investigate the mechanisms underlying its regulation of LPS-induced ALI inflammatory responses, this study initially conducted a comprehensive analysis using relevant databases to identify previously unexplored target genes closely associated with sepsis-related ALI. SLC16A10 emerged as our candidate gene, and this relationship was experimentally validated through a luciferase reporter assay, revealing that miR-21-5p may exert its regulatory role in inflammation by targeting SCL16A10.

SLC16A10 is a member of the SLC16 family, located on 6q21-q22, consisting of 6 exons with a length of 136 kb. It primarily participates in the transport of thyroid hormone (TH) and aromatic amino acids and is a unidirectional amino acid transporter that does not depend on Na^+^ and protons^36,37^. Under normal circumstances, SLC16A10 is expressed at low levels in cells, but its mRNA levels in the intestinal mucosa increase in response to LPS stimulation^38,39^. Studies have indicated that SLC16A10 plays a role in mouse T cell-mediated immune responses, and its upregulation is associated with systemic inflammatory diseases, suggesting that elevated SLC16A10 expression may be related to changes in immune homeostasis and spontaneous inflammation^28^. Additionally, research has reported that high expression of SLC16A10 reduces the tryptophan content in cells, and tryptophan has a strong protective effect on lung tissues in LPS-induced ALI mice, reducing the expression of pro-inflammatory cytokines^40^.

Our experimental results demonstrate that downregulating SLC16A10 expression significantly inhibits the expression of inflammatory factors in LPS-induced A549 cells. This, in conjunction with previous research findings, confirms the pro-inflammatory role of SLC16A10. Finally, through the simultaneous downregulation of both miR-21-5p and SLC16A10, we observed that the downregulation of SLC16A10 reversed the inhibitory effect of miR-21-5p on the expression of inflammatory factors. This result suggests that the low expression of miR-21-5p may promote the inflammatory response in ALI through downstream target SLC16A10.

**Figure 8:**
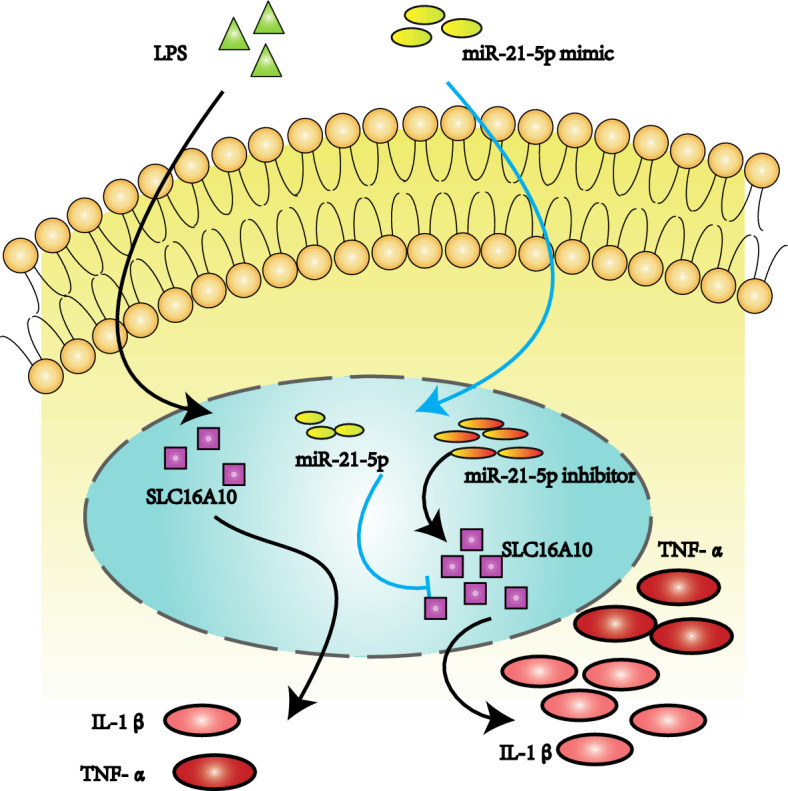
Schematic diagram of miR-21-5p action mechanism.

In summary, our research strongly suggests that miR-21-5p plays a crucial negative regulatory role in the inflammatory response of acute lung injury, primarily by targeting SLC16A10 to mitigate the expression of inflammatory factors (Fig. 8). This discovery provides compelling evidence for a better understanding of the pathogenesis of ALI and the development of potential therapeutic strategies. However, further research is needed to elucidate the detailed mechanisms of miR-21-5p and SLC16A10 in ALI and their potential applications in clinical treatment.

### Supplementary Information

Supplementary Information 1.

Supplementary Information 2.

## Data Availability

The authors confirm that the data supporting the findings of this study are available within the article.
