# Supplementary material for: MiR-21-5p modulates LPS-induced acute injury in alveolar epithelial cells by targeting SLC16A10
Source: Sci Rep. 2024 May 15;14:11160. doi: 10.1038/s41598-024-61777-x (PMC11096310; doi:10.1038/s41598-024-61777-x)

Fig1B				
IL-1β	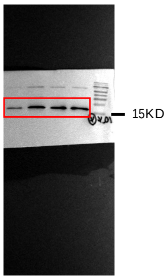	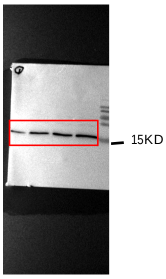	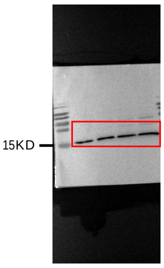	17KD
TNF-α	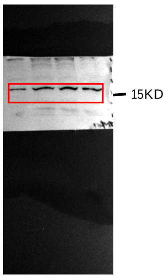	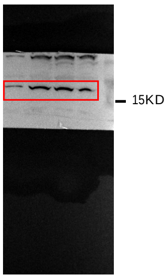	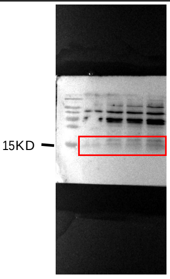	16KD
GAPDH	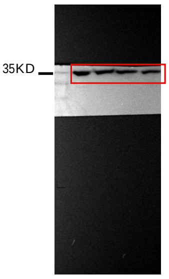	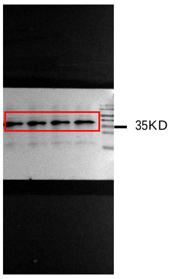	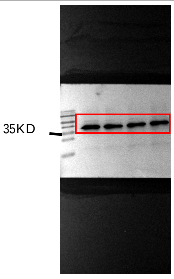	36KD

All images in the above figure represent three replicates for the corresponding gene, with the respective marker sizes based on the Thermo Fisher marker guide, as shown in the right-side image. The groupings for all images are ordered from left to right as GC,LPS 6h,LPS12h,LPS24h

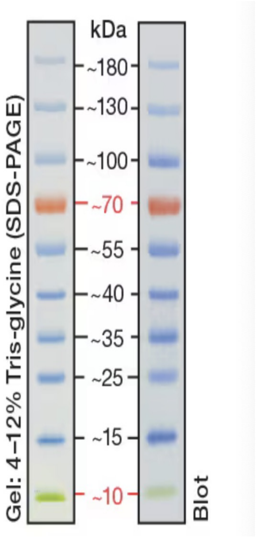

Fig3D				

IL-1β	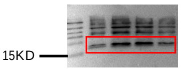	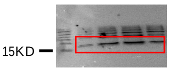	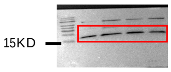	17KD
TNF-α	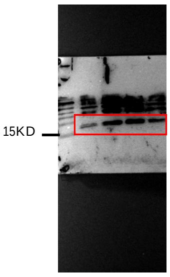	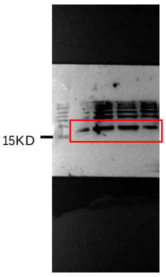	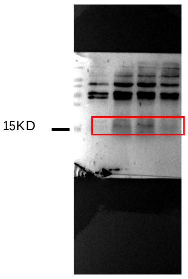	16KD
GAPDH	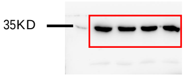	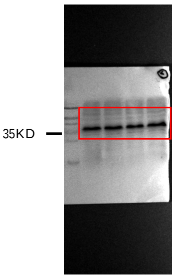	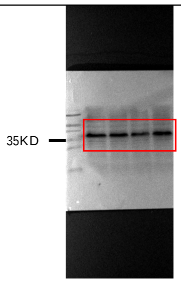	36KD

All images in the figure above represent triplicate images for the corresponding gene, with the specific marker sizes indicated by the Thermo Fisher marker, as shown in the image on the right. The order of group information for all images, from left to right, is control， LPS ,LPS+NC mimic,LPS+miR-21-5p mimic.

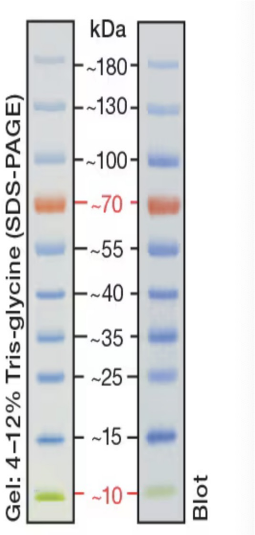

Fig5D				
SLC16A10	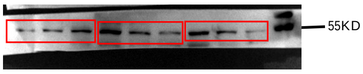			55KD
GAPDH	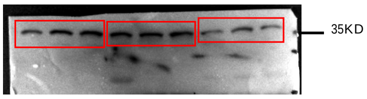			36KD
Fig5F				
SLC16A10	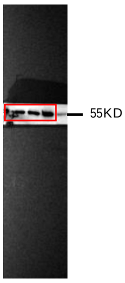	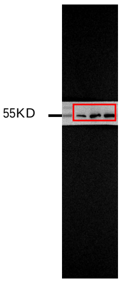	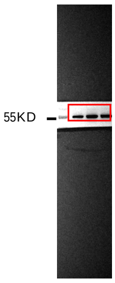	55KD
GAPDH	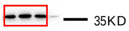	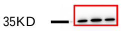	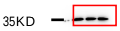	36KD

All images in the figure above represent triplicate images for the corresponding gene. The specific marker sizes are based on the Thermo Fisher marker guide, as shown in the image on the right. The group information for Fig. 5D and 5F is control, NC mimic, miR-21-5p mimic from left to right.

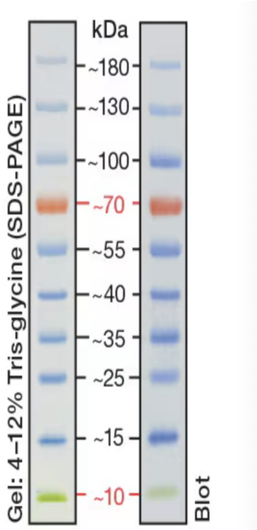

Fig6B				
SLC16A10	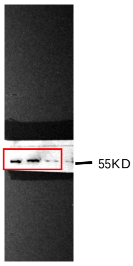	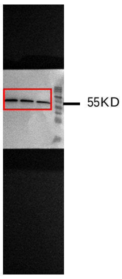	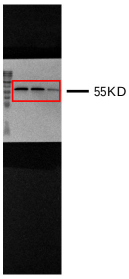	55KD

GAPDH	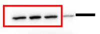 35KD	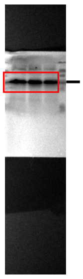 35KD	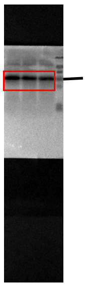 35KD	36KD

All images in the figure above represent triplicate images for the corresponding gene. The specific marker sizes are based on the Thermo Fisher marker guide, as shown in the image on the right. The group information for Fig. 6B is control， NC siRNA,si-SLC16A10

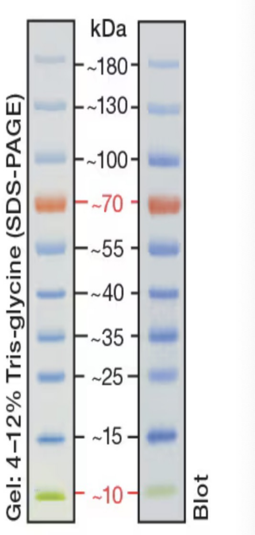

Fig6D				

IL-1β	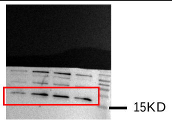	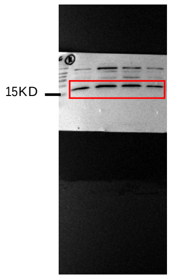	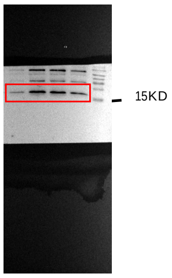	17KD
TNF-α	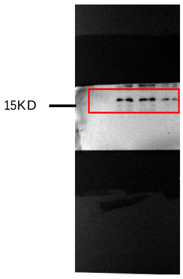	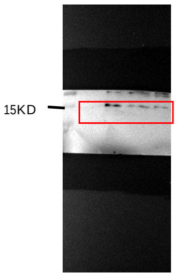	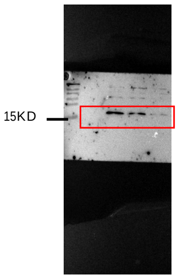	16KD
GAPDH	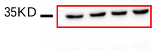	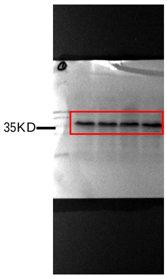	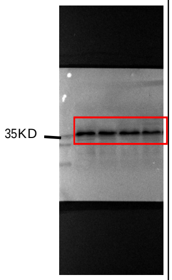	36KD

All images in the figure above represent triplicate images for the corresponding gene. The specific marker sizes are based on the Thermo Fisher marker guide, as shown in the image on the right. The group information for Fig. 6D is control,LPS,LPS+ NC siRNA,LPS+si-SLC16A10

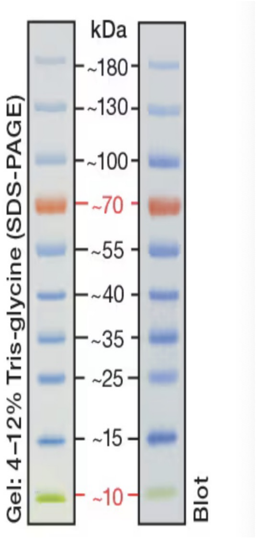

Fig7B				
SLC16A10	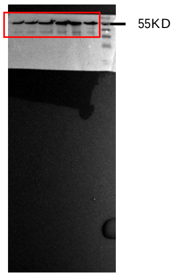	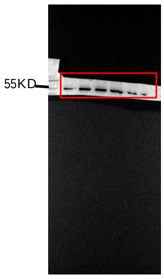	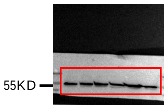	55KD
TNF-α	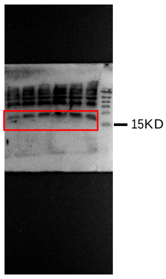	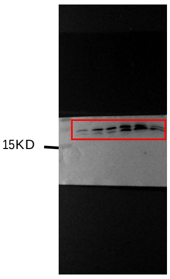	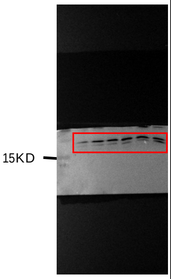	16KD
IL-1β	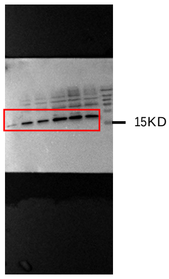	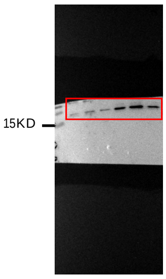	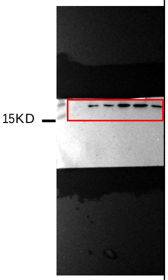	17KD
GAPDH	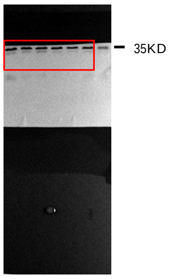	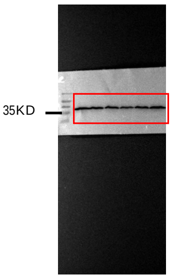	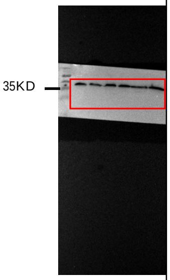	36KD

All images in the figure above represent triplicate images for the corresponding gene. The specific marker sizes are based on the Thermo Fisher marker guide, as shown in the image on the right. The group information for Fig. 7B is control,LPS,LPS+NC inhibitor,LPS+miR-21-5p inhibitor,LPS+miR-21-5p inhibitor+NC siRNA,LPS+miR-21-5p inhibitor+si-SLC16A10

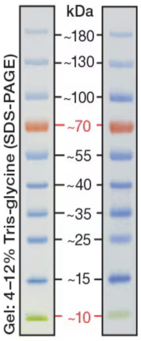

Supplement: Supplementary file 2 — Supplementary Information 2. [file 41598_2024_61777_MOESM2_ESM.pdf]
